# The Role of Limited Emotion Regulation Strategies on Nonsuicidal Self-injury and Suicide Attempts among Chinese Adolescents: A Network Analysis Based on Jiangxi Province

**DOI:** 10.34133/hds.0195

**Published:** 2026-02-03

**Authors:** Hao Xu, Xuejing Xu, Yan Chen, Wei Qu, Yunlong Tan, Zhiren Wang, Yanli Zhao, Shuping Tan, Dianying Liu

**Affiliations:** ^1^ Peking University Huilongguan Clinical Medical School, Beijing Huilongguan Hospital, Beijing 100096, China.; ^2^ North China University of Science and Technology, Tangshan 063210, China.; ^3^ Temple University, Philadelphia, PA 19122, USA.; ^4^ Ganzhou City Key Laboratory of Mental Health, The Third People’s Hospital of Ganzhou City, Ganzhou 341000, China.

## Abstract

**Background:** Nonsuicidal self-injuries (NSSIs) are an important contributing factor to adolescent suicide, and various shared factors influence the risk of both NSSIs and suicide attempts (SAs). Both are important predictors of suicide and are part of a continuum of suicidal behaviors. Further exploration of the relationship between adolescent NSSI and SA may facilitate suicide prevention efforts. **Methods:** An online survey was conducted among 9,140 participants. Network analysis methods were used to explore expected influence (EI), bridge expected influence (BEI), edge weights, and differences between adolescents that have and have not attempted suicide (NSSI-SA and NSSI-NoSA, respectively). **Results:** Of the 9,140 participants, 7,030 completed the questionnaire, yielding a participation rate of 76.91%. Participants with at least one NSSI were retained, with 2,496 (35.50%) included in the network analysis. The strongest EI node for both networks was “emotion regulation strategies” (*E* = 1.389 and 1.393), and that for BEI was “personal distress” (Interpersonal Reactivity Index—personal distress; *E* = 0.497 and 0.492). Network comparisons revealed significant differences in NSSI 4 (“intentionally hitting walls, tables, and other hard objects”; *E*_(Δ)_ = −0.384, *P* < 0.001), significant differences in BEI with regard to “perspective taking” (Interpersonal Reactivity Index—perspective taking; *E*_(Δ)_ = −0.215, *P* < 0.001), and significant differences in edge weights between NSSI 4 and NSSI 5 (“intentionally hurting oneself by hitting with a fist, palm, or hard object”; *E*_(Δ*r*)_ = −0.173, *P* < 0.001). **Conclusions:** Our study suggests that interventions in the form of emotion regulation strategies can alleviate symptoms throughout the entire network. Attention should be paid to instances when NSSI 4 and NSSI 5 behaviors co-occur frequently.

## Introduction

Adolescent suicide remains a critical global public health concern, being a leading cause of nonnatural mortality [1,2]. While suicide attempts (SAs) directly contribute to fatal outcomes, nonsuicidal self-injury (NSSI)—defined as deliberate, repetitive bodily harm without lethal intent, often serving emotion regulation [3–5]—represents a distinct yet interconnected behavioral spectrum. However, SA and NSSI are conceptually distinct: SA is characterized by a desire to end one’s life, while NSSIs are intended to inflict harm without lethal intent. SA directly endangers an individual’s life, whereas NSSIs primarily damage body tissues. Notably, the worldwide incidence rate of NSSI in children and adolescents is approximately 19.5%, whereas in Chinese adolescents (13 to 18 years of age), the rate reaches 27.4% [6,7]. This disparity may be rooted in China’s sociocultural context: academic pressure, family dynamics (e.g., parental migration in rural communities), and stigma against mental health disclosure may uniquely amplify vulnerability in Chinese adolescents.

Although NSSI and SA are conceptually distinct, their comorbidity is well-documented. A growing number of studies have shown a strong correlation between NSSI and SA in adolescents [8] and have highlighted their complex relationship, influenced by multiple factors [9,10], such as affective disorder, cognitive factors, and behavioral problems. Depression, a core affective disorder, bridges NSSI and SA through difficulties in emotion regulation and is associated with symptoms including prolonged sadness, guilt, loss of interest, significant weight loss or gain, increased or decreased appetite, fatigue, memory loss, anxiety, and sleep problems [11]. It may also manifest as irritability or through other physical symptoms (such as abdominal pain, chest pain, and headache). Difficulties in emotion regulation may drive physical pain-seeking via NSSI to alleviate negative emotion, which may aggravate negative emotion that may also escalate risk toward SA. Concurrently, comorbid behavioral disorders like mobile phone addiction [12] compound risk through dual pathways: it weakens the real interpersonal support system through social avoidance behavior, and instant thrill-seeking characteristics may form a reinforcement cycle. These factors may be mediated by cognitive deficits in emotion regulation and empathy [13,14]. Gender, residence, and family structures are also early risk factors affecting adolescents’ NSSI and SA [15–17]. Given the complex relationship between NSSI and SA, further exploration could enrich theoretical foundations in suicide research and inform clinical prevention, diagnosis, and intervention.

Traditional diagnostic frameworks (e.g., DSM-5 and ICD-11) classify NSSI and SA as separate entities [18,19]. Specifically, DSM-5 added NSSI as a separate behavioral disorder in 2017, defining criteria such as frequency (i.e., ≥5 episodes within 12 months) while excluding accidental self-harm, suicidal behavior, and culturally accepted practices (e.g., tattoos and piercings). However, these classifications have advanced the field but faced challenges regarding reliability, validity, and clinical utility [20,21]. Their “common cause” model may inadequately explain comorbidity and dynamic interactions. In recent years, with the continuous advancement of diagnostic and statistical methods [22,23], network theory offers an alternative perspective, conceptualizing psychopathology as interconnected symptom networks characterized by central nodes and edges [24]. Theoretically, high-centrality nodes may play important roles in the network and are worthy intervention targets [25]. Clarifying these interactions may elucidate mechanisms underlying adolescent NSSI and SA and guide targeted clinical intervention strategies [26]. Recent studies have applied network analysis to provide valuable insights into NSSI and SA in adolescents [27,28], yet a comprehensive theoretical framework remains underdeveloped. Although NSSI is a strong predictor of SA, the mechanisms linking both behaviors remain unclear, and network analysis applications are still limited.

Therefore, this study employs network analysis to compare network metrics between Chinese adolescents’ NSSI with SA and NSSI without SA and identify central symptoms and bridge pathways (e.g., depression, difficulties in emotion regulation, phone addiction dimensions, and NSSI types) driving comorbidity. This study aims to supplement theoretical frameworks for reducing SA risk and improving clinical interventions.

## Methods

### Participants

Between September and December 2022, an online survey was carried out, involving 9,140 students from 3 high schools in a city in Jiangxi Province, China. The participants were required to complete a mobile-based questionnaire, which gathered demographic and psychological data. The inclusion criteria were 12 to 18 years old, with no gender restrictions, and voluntary participation in the study. In order to prevent data contamination, our study was completed by the school’s teachers and 3 major researchers, and training was provided to the teachers involved in the research. The teacher distributed the QR code of the questionnaire to the class, each IP address can be accessed only once, and the researchers explained the relevant questions to the teenagers and their guardians. The questionnaire was answered by mobile devices in the electronic case report form (eCRF) system of Beijing Huilongguan Hospital [29]. Participants signed informed consent online, where the schoolteachers obtain the informed consent of their guardians online. If they did not agree to participate in the study, the system would automatically quit and their information would not be saved. For the privacy of the participants, the data are uniformly stored in the eCRF system of Beijing Huilongguan Hospital. This study was approved by the Ethics Committee of the Beijing Huilongguan Hospital (approval number 2021-24-Ke) and strictly followed the principles of the Declaration of Helsinki.

### Measures

#### Suicide attempts

Participants were invited to answer 2 questions [5], namely, “Have you ever had a suicide attempt in your lifetime?” and “Have you had any suicide attempts in the past 12 months?” If the response to either of these items was “yes”, the individual was categorized as having a history of SAs.

#### Nonsuicidal self-injury

The Adolescent NonSuicidal Self-Injury Assessment Questionnaire (ANSAQ) was used to assess participants’ NSSI [30]. The questionnaire contains 12 questions rated on a 5-point Likert scale, with each item corresponding to a specific self-injurious behavior. The severity of NSSI was correlated with the total score. For example, item 1 is deliberately pinching oneself, and item 2 is deliberately scratching oneself, and so on.

#### Depression

We used the Patient Health Questionnaire-9 (PHQ-9) to evaluate participants’ depressive states [31]. The questionnaire contains 9 items rated on a 4-point Likert scale (e.g., “lack of enthusiasm or interest when doing things”). Depression severity increased in line with PHQ-9 score increases.

#### Emotion regulation

The Chinese version of the Difficulties in Emotion Regulation Scale (DERS) was used to determine adolescents’ degree of difficulty in regulating their emotions [32]. The 36 items of the DERS were rated on a 5-point Likert scale (e.g., “I am clear about my feelings” and “I pay attention to how my fee”). Higher total scores were indicative of more difficulties in emotional regulation. The DERS comprises 6 dimensions: lack of emotional awareness (awareness), which reflects a diminished capacity to recognize and monitor emotional states, often manifested as delayed identification or misinterpretation of one’s affective experiences; lack of emotional clarity (clarity), which characterizes difficulties in differentiating and articulating distinct emotional states, leading to vague or undifferentiated self-reports of feelings; nonacceptance of emotional responses (nonacceptance), which captures tendencies to judge emotions as unacceptable or irrational, particularly negative affective states perceived as threatening or uncontrollable; impulse control difficulties (impulse), which indicate an impaired ability to inhibit maladaptive behaviors during emotional arousal, resulting in impulsive actions for immediate relief despite adverse consequences; engaging in goal-directed behavior (goals), which assesses disruptions in maintaining task-oriented focus and executing planned actions when experiencing intense emotions; and limited access to emotion regulation strategies (strategies), which denote a restricted repertoire of adaptive coping mechanisms and insufficient knowledge about effectively managing emotional distress.

#### Tendency toward mobile phone addiction

The participants’ mobile phone addiction tendencies were assessed using the Chinese version of the Mobile Phone Addiction Tendency Scale (MPATS) [33]. The questionnaire contains 16 items rated on a 5-point Likert scale. The MPATS primarily focuses on the subjective experience of internal cognitive processes and social interactions among mobile phone users. It consists of 4 dimensions: withdrawal symptoms (negative physical or psychological reactions to not participating in mobile phone activities), salient behavior (the use of mobile phones occupies the center of thought and action), social comfort (the role of mobile phones in interpersonal communication), and mood changes (changes in mood caused by mobile phones). A higher total score indicates greater severity of mobile phone addiction. For example, item 1 is, “If I haven’t brought my mobile phone for a while, I will immediately check if there are any text messages or missed calls.”

#### Interpersonal reactivity

The Chinese Interpersonal Reactivity Index (C-IRI) was used to assess adolescents’ empathic capacity in interpersonal communication [34], with the purpose of evaluating their ability to establish emotional and cognitive connections with others. The scale consists of 28 items measured on a 5-point Likert scale (e.g., “For those who are less fortunate than me, I often have a feeling of tenderness and care”). Higher total scores indicate greater empathic abilities. The scale has 4 factors: perspective taking (PT), which measures understanding and empathy toward others’ perspectives in real-life situations; empathic concern (EC), which assesses sympathy and concern for others; fantasy (FS), which examines emotional immersion in fictional characters; and personal distress (PD), which measures emotional reactions to others’ misfortunes.

### Statistical analysis

#### Participant characteristics

The initial sample size was 9,140. To eliminate the interference of extreme or abnormal values and ensure the accuracy and reliability of the data, we removed samples with response times exceeding 3 standard deviations from the mean, resulting in 7,030 remaining responses. This is because such deviations could indicate either too many interruptions or too much decisiveness in the answering process, thus affecting the reliability of the data. We subsequently eliminated entire responses from participants that were associated with one or more missing values in their answers. After removing the samples with missing values, 6,956 samples were left; of these, 2,496 adolescents who reported a history of NSSI were ultimately included in the analysis. Participants were divided into 2 groups based on their history of SAs (NSSI with no SA [NSSI-NoSA] and NSSI with SA [NSSI-SA]). As each scale had different scoring rules, we transformed the original data into *z*-scores.

#### Network estimation

We performed a network analysis using 12 items from the NSSI Behavior Scale, 9 items from the PHQ-9, 4 factors from the IRI, 6 factors from the DERS, and 4 factors from the MPATS. We used the estimateNetwork with the EBICglasso default setting in R package bootnet in order to fit regularized partial correlation networks separately for the NSSI-NoSA and NSSI-SA groups [35]. The R package qgraph was used to assess the expected influence (EI) of nodes. A higher EI indicates greater importance of the node [36]. The bridge expected influence (BEI) was calculated using the R package networktools [37]. Compared to nodes with a low BEI, nodes with a high BEI signify a higher risk of information transmission between communities. In network analysis, communities are typically defined as groups of nodes that demonstrate denser connections within the group than with nodes outside the group, reflecting modular structures in the network. The top 20% of nodes with the highest BEI were identified as bridge nodes [38].

To ensure the stability and accuracy of the estimated networks, we employed the R package bootnet with a bootstrap parameter set to 1,000 (95% confidence intervals [CIs]). We used the R package NetworkComparisonTest to compare the differences between the NSSI-NoSA and NSSI-SA groups. This involved conducting 1,000 permutations to examine network invariance, global strength, edge, and centrality invariances. Paired tests were 2-sided with a significance threshold set at 0.05. Holm–Bonferroni (BH) correction was applied to adjust for multiple comparisons [39]. The averageLayout function was used to ensure that the 2 networks were displayed with an average layout [35].

Data preparation, initial analysis, model development, and visualization were performed using SPSS Statistics 27 and R 4.3.0.

## Results

### Participant characteristics

Table 1 displays the characteristics of the 2,496 participants. Among 23.03% of participants with a history of SA, 69.60% were female and the median age was 16 years. The results of the independent sample *t* tests for each scale item can be found in Table S1. The characteristics of 6,959 participants can be found in Table S2 or by referring to our previous paper [5].

**Table 1.** Characteristics of participants. Data shown as *N*(%) and median(Q1, Q3).

Characteristic	NSSI-NoSA	NSSI-SA	*z*/χ^2^	*P* value
	*N* = 1,921(76.96%)	*N* = 575(23.03%)		
	*N* (%)/median(Q1, Q3)	*N* (%)/median(Q1, Q3)		
Gender(female) ^a^	975(50.80%)	400(69.60%)	63.29	<0.001
Age ^b^	16(15, 17)	16(15, 16)	−1.64	0.10
Height ^b^	165(160, 170)	163(158, 168)	−6.57	<0.001
Weight ^b^	55(48, 70)	53(47, 78)	−1.42	0.16
One-child family ^a^	84(4.37%)	29(5.04%)	0.46	0.50
Order of birth ^a^			6.995	0.14
First	718(37.38%)	225(39.13%)		
Second	835(43.47%)	266(46.26%)		
Third	229(11.92%)	55(9.57%)		
Fourth	91(5.05%)	18(3.13%)		
Fifth	42(2.19%)	11(1.91%)		
Type of residence ^a^			4.50	0.11
Downtown	203(10.57%)	66(11.48%)		
Suburb	506(26.34%)	174(30.26%)		
Rural area	1,212(63.09%)	335(58.26%)		
Way of parenting ^a^			2.03	0.73
I	899(46.80%)	254(44.17%)		
II	380(19.78%)	117(20.35%)		
III	413(21.50%)	135(23.48%)		
IV	105(5.47%)	35(6.09%)		
V	124(6.46%)	34(5.91%)		
PHQ-9 ^b^	8(5, 12)	12(8, 17)	−13.79	<0.001
IRI ^b^	90(82, 97)	94(87, 102)	−7.67	<0.001
MPATS ^b^	44(34, 52)	50(41, 59)	−9.81	<0.001
DERS ^b^	90(82, 102)	102(90, 117)	−13.19	<0.001
NSSI behavior ^b^	3(1, 6)	8(3, 16)	−15.49	<0.001

NoSA, no suicide attempt; SA, suicide attempt; I, living with parents; II, one of the parents is absent periodically; III, living with grandparents without parents; IV, single parent family; V, others; PHQ-9, Patient Health Questionnaire-9; IRI, Interpersonal Reactivity Index; MPATS, Mobile Phone Addiction Tendency Scale; DERS, Difficulties in Emotion Regulation Scale; NSSI, Nonsuicidal Self-Injury Assessment Questionnaire

^a^ Chi-squared test.

^b^ Mann–Whitney *U* test.

### Network structure

**Fig. 1.**
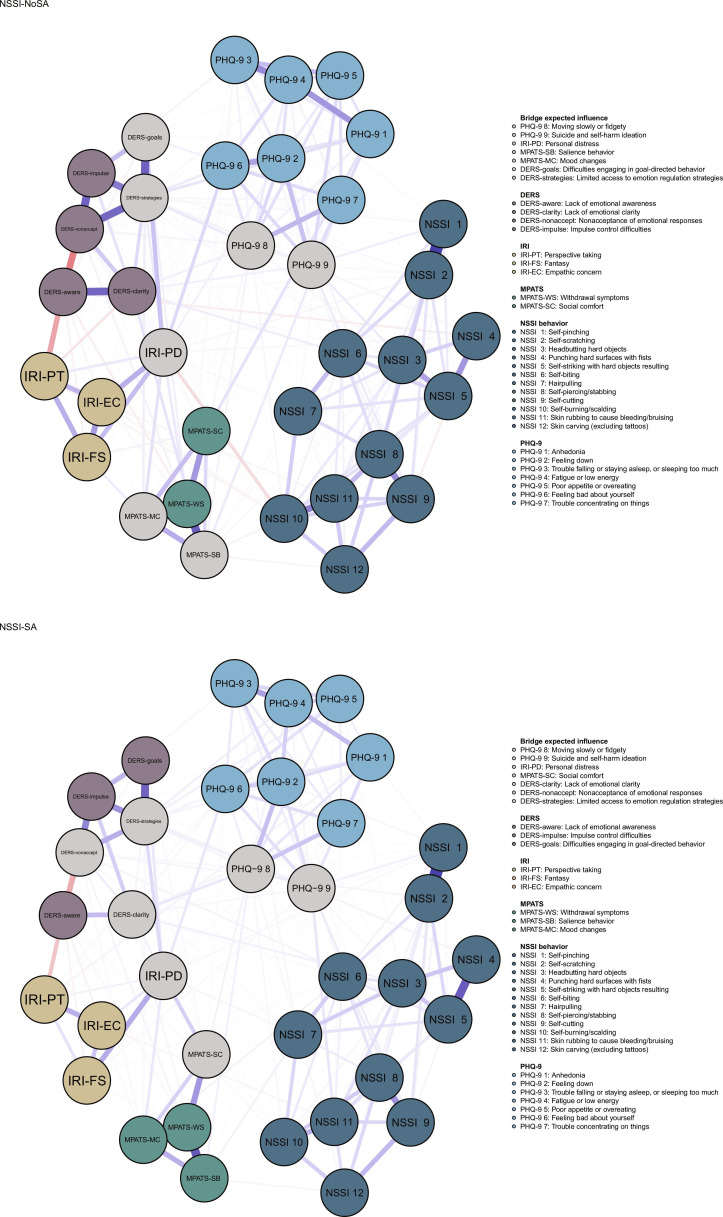
Network structures of NSSI-NoSA and NSSI-SA.

Figure 1 illustrates the network structures of the NSSI-NoSA and NSSI-SA groups; in both networks, a total of 43.36% (258 of 595) and 38.82% (231 of 595) of the edges were nonzero, respectively. The average edge weight for both networks was 0.024, indicating a comparable network density between the NSSI-NoSA and NSSI-SA groups. In Fig. 1, the connections between nodes are represented by edges, with thicker edges representing stronger weights. The blue and red lines represent positive and negative correlations, respectively. The nodes are predominantly distributed within specific communities.

### Node centrality and network stability

We estimated the stability of the node EI and BEI in the NSSI-NoSA and NSSI-SA groups, as shown in Figs. S1 and S2, respectively. The centrality stability coefficient for the NSSI-NoSA group was 0.75 for both EI and BEI. Similarly, for the NSSI-SA group, the centrality stability coefficient was 0.67 for both EI and BEI. These values exceed the recommended minimum of 0.25 and surpassed the preferable threshold of 0.5, which indicates the acceptable reliability of the centrality indices (Figs. S1 and S2) [40]. The CIs for the edges in both networks ranged from small to moderate, indicating reliable accuracy (Figs. S3 and S4). Table 2 presents the top 10 nodes with the highest EI and the top 20% of nodes with the highest BEI in both networks (Figs. S5 and S6). The nodes with the highest EI in NSSI-NoSA and NSSI-SA are both strategies (*E* = 1.389 and *E* = 1.393), while the nodes with the highest BEI are both IRI-PD nodes (*E* = 0.497 and *E* = 0.552).

**Table 2.** The node centrality for networks: NSSI-NoSA and NSSI-SA

NSSI-NoSA				NSSI-SA			
EI nodes	*E*	BEI nodes	*E*	EI nodes	*E*	BEI nodes	*E*
DERS strategies	1.389	IRI-PD	0.497	DERS strategies	1.393	IRI-PD	0.552
NSSI 2	1.176	DERS strategies	0.397	NSSI 2	1.189	DERS strategies	0.471
NSSI 8	1.111	MPATS-SB	0.327	NSSI 5	1.121	PHQ-9 9	0.428
MPATS-WS	1.089	DERS-goals	0.304	PHQ-9 4	1.046	DERS-clarity	0.391
NSSI 5	1.070	PHQ-9 9	0.300	MPATS-WS	1.033	PHQ-9 8	0.328
MPATS-MC	1.036	MPATS-MC	0.272	NSSI 3	1.000	MPATS-SC	0.304
PHQ-9 4	1.002	PHQ-9 8	0.243	PHQ-9 8	0.999	DERS-nonaccept	0.249
MPATS-SB	0.958			PHQ-9 2	0.999		
PHQ-9 2	0.955			NSSI 1	0.996		
PHQ-9 6	0.951			NSSI 8	0.980		

EI nodes, top 10 expected influence nodes; BEI nodes, top 20% of bridge expected influence nodes; *E*, value of expected influence

### Network comparison test

Differential analysis was performed on the 2 networks. First, a network invariance test revealed a significant difference in the average degree between the 2 networks (*M* = 0.173, *P* = 0.002), indicating that at least one edge differed significantly between them. The global strength invariance test showed that overall node connectivity, represented by the sum of the degrees of all nodes in the networks, did not differ significantly between the NSSI-NoSA and NSSI-SA groups (*S* = 16.498 and 16.080, respectively; *P* = 0.136). Table 2 presents the results of the centrality invariance test. After applying the BH correction, only NSSI 4 remained significant (Table 3).

**Table 3.** Centrality comparison for NSSI-NoSA and NSSI-SA

Nodes	NSSI-NoSA	NSSI-SA	*E*_(Δ)_	*P* value
EI nodes				
NSSI-4	0.290	0.674	−0.384	<0.001*
NSSI-1	0.857	0.996	−0.140	0.011
NSSI-3	0.870	1.000	−0.130	0.044
NSSI 8	1.111	0.980	0.131	0.048
PHQ-9 8	0.871	0.999	−0.128	0.048
BEI nodes				
IRI-PT	−0.289	−0.074	−0.215	<0.001*
DERS-goals ^a^	0.304	0.138	0.166	0.003
DERS-clarity ^b^	0.235	0.391	−0.157	0.034
MPATS-WS	0.118	−0.013	0.131	0.042
PHQ-9 9 ^a^^,^^b^	0.300	0.428	−0.128	0.025
PHQ-9 7	0.198	0.076	0.122	0.038
MPATS-SB ^a^	0.327	0.211	0.116	0.040

*, Holm–Bonferroni correction, adjusted *P* < 0.05; NSSI-4 (adjusted *P* = 0.035), IRI-PT (adjusted *P* = 0.035). EI nodes, network comparison results were significant for expected influence nodes; BEI nodes, network comparison results were significant for bridge expected influence nodes

^a^ Top 20% of bridge expected influence nodes in NSSI-NoSA.

^b^ Top 20% of bridge expected influence nodes in NSSI-SA.

The comparison of BEI between the 2 networks revealed significant differences in 7 bridge nodes, as well as in IRI-PT (*E*_(Δ)_ = −0.215, *P* < 0.001; Table 3). Among the top 20% of BEI values, 4 nodes showed significant differences. After applying the BH correction, only IRI-PT remained significant.

The edge-invariance test showed significant differences in 44 edges between the 2 networks. Table 4 presents the top 10 significantly different edge weights, along with the test statistics and significance levels. However, following the application of the BH correction, none of the differences remained significant.

**Table 4.** Ten largest significant edge-weight differences in NSSI-NoSA and NSSI-SA

Edges	Edge weights		*E*_(Δ*r*)_	*P* value
	NoSA	SA		
NSSI 4–NSSI 5	0.232	0.405	−0.173	<0.001
IRI-PD–MPATS-SC	0.022	0.148	−0.126	<0.001
NSSI 9–NSSI 10	0.123	0.003	0.121	0.003
IRI-EC–IRI-PD	0.188	0.081	0.107	0.002
MPATS-SB–MPATS-SC	0.107	--	0.107	0.009
DERS-aware–DERS-clarity	0.307	0.201	0.106	0.005
NSSI 1–NSSI 2	0.517	0.614	−0.097	0.002
PHQ-9 5–PHQ-9 9	0.102	0.010	0.091	0.021
PHQ-9 5–PHQ-9 6	0.013	0.105	−0.091	0.003
IRI-PT–IRI-FS	0.188	0.100	0.088	0.024

--, partial correlation not detected in the regularized network

## Discussion

We conducted a network analysis of adolescents with NSSI, with and without a history of SA, to compare the differences in their networks. The network structures of the NSSI-NoSA and NSSI-SA groups showed no significant differences, suggesting shared influencing factors between these behaviors.

Our study found that among adolescents exhibiting NSSI, females reported a higher proportion of SA, which is consistent with the findings of Wang et al. [17]. The higher SA prevalence among rural adolescents aligns with findings from mainland China [41], potentially reflecting unique socioeconomic pressures in Chinese rural settings (e.g., parental migration for work). However, our study did not replicate the association between SA and parental absence reported [42], possibly due to cultural differences in family dynamics. Furthermore, the association between SA and birth order observed in our study supports the findings of Easey et al. [15]. The 2 studied groups of adolescents were also significantly different in terms of PHQ-9, IRI, MPATS, and DERS scores as well as NSSI behaviors. This might be an indicator of the greater severity of these mental health aspects of adolescents with NSSI who also have a history of SA.

The absence of statistically significant differences in global network structure between the NSSI-NoSA and NSSI-SA groups provides potential support for the hypothesis that NSSI and SA in adolescents may share overlapping etiological pathways [43]. In both the NSSI-NoSA and NSSI-SA groups, the “limited strategies” dimension exhibited the highest EI, a pattern consistent with recent network analyses of emotion regulation profiles [27]. This centrality may primarily reflect strong within-construct correlations among DERS dimensions (e.g., tight connections between “limited strategies” and other emotion regulation deficits) rather than cross-domain influences on external symptom clusters. While the observed centrality of “limited strategies” aligns with established associations between emotion regulation deficits and adolescent NSSI [44,45], these findings tentatively suggest that interventions targeting this dimension could potentially modulate network dynamics. Adolescence represents a sensitive period for emotion regulation development [46] and improving adaptive strategy use might indirectly affect comorbid symptoms through intraconstruct mechanisms. Evidence suggests that NSSI are more prevalent during adolescence, SA during late adolescence, and completed SAs occur more frequently in mid-adulthood [47]. Therefore, positive intervention in the form of emotion regulation strategies during adolescence may help prevent future suicide risk. Although medication plays a positive role in alleviating the symptoms of emotional disorders in adolescents, cognitive-level treatments and education should be focused on as well. Existing research indicates that cognitive behavioral therapy and dialectical behavioral therapy for adolescents can modify emotion regulation strategies in adolescents and are optimal approaches to treating adolescents with NSSI and SA [48,49]. Additionally, schools play a crucial role in introducing preventive measures against adolescent suicide. Furthermore, implementation of suicide intervention measures in primary and secondary schools can effectively reduce SAs among adolescents [50].

The strongest BEI node in both the NSSI-NoSA and NSSI-SA networks was IRI-PD, indicating the importance of negative emotional empathy in interpersonal interactions among adolescents. IRI-PD is closely related to goals in the DERS community node in the NSSI-NoSA network, which suggests the influence of PD on adolescents’ ability and motivation to pursue and achieve important goals when facing negative emotions; this may be related to behavioral issues such as academic decline and disengagement from school [12]. In the NSSI-SA network, IRI-PD is the most closely connected to MPATS-SC, indicating that PD may have an impact on the role of mobile phone use in interpersonal interactions among adolescents, emphasizing the social aspect of phone use and the potential for mobile phone addiction; previous research has also revealed a significant correlation between IRI-PD and addictive behaviors [51].

Comparing the centrality between the 2 networks, the IRI-PT in the NSSI-NoSA network (*E* = −0.289) was significantly higher than that in the NSSI-SA network (*E* = −0.074). The ability to engage in perspective taking may play a more positive role in alleviating the severity of other nodes in the NSSI-NoSA network, leading to greater intervention benefits, which aligns with our expectations. The importance of the IRI-PD and IRI-PT nodes in terms of the BEI may partially explain the significant role of interpersonal relationships in adolescent NSSI and SA [13]. Adolescent mobile phone addiction is associated with various emotional disorders and maladaptive behaviors [52], and our BEI analysis also indicates that MPATS-SB and MPATS-MC in the NSSI-NoSA group, and MPATS-SC in the NSSI-SA group, are among the top 20% of nodes, with significant differences observed for MPATS-SB. This finding suggests the need for caution regarding the spread of mobile phone addiction among adolescents to other community nodes. Another finding was that when comparing the top 20% bridging potential influence nodes, the BEI of the PHQ-9 (suicidal ideation) node in the NSSI-SA group was significantly higher than that in the NSSI-NoSA group. This indicates that suicidal ideation plays a significant role in maintaining the NSSI-SA network and has a greater possibility of spreading to other communities (symptom clusters).

Self-cutting is the most frequent form of NSSI encountered [53]. A recent study found a high prevalence rate (69.2%) of “slamming into walls or other objects” [54]. Our network comparison results indicate significant differences in the EI of NSSI 4 (“intentionally punching walls, desks, windows, floors, and other hard objects”) between the NSSI-SA and NSSI-NoSA groups, with the EI of the NSSI-SA network being significantly higher than that of the NSSI-NoSA network. Furthermore, we observed subtle differences in edge weights between the 2 networks, whereas the edge weights between the NSSI 4 and NSSI 5 nodes in the NSSI-NoSA and NSSI-SA networks were significantly different. Specifically, the connection between “intentionally punching walls, desks, and other hard objects” and “intentionally hitting oneself with fists, slaps, or harder objects” differs significantly. The edge weights of NSSI 4 and NSSI 5 in the NSSI-SA group were significantly higher than those in the NSSI-NoSA group, being twice as high as those in the NSSI-NoSA group. This may be related to higher levels of impulsivity and more aggressive behaviors exhibited by NSSI-SA adolescents [55]. Therefore, clinicians must pay attention to NSSI adolescents who exhibit both NSSI 4 and NSSI 5 behaviors, as they may be at greater risk for future SAs. We hope that through the network analysis method, this comprehensive global thinking may bring new theoretical insights and clinical applications for understanding NSSI and SA among adolescents. In future research, we plan to employ a longitudinal design to conduct long-term follow-up investigations of adolescents with NSSI to explore the factors that contribute to SAs.

Our study had some limitations. First, the study was exploratory in nature, and clinical interpretations should be made with caution. Second, this study adopted a cross-sectional design, using nonclinical samples. Additional information about the sociodemographic characteristics of the sample was not considered. The data were collected during the late stages of the coronavirus disease 2019 pandemic and relied on self-report measures, which may have led to an overestimation of the number of adolescents with a history of SA as a result of the limitations of the research tools. We did not include the total score of the scale in this network analysis; in future research, we will refine the comparison of the differences between item-level and overall hierarchical networks. Future research may focus on elaborating these relationships to provide a more comprehensive perspective on the dynamics of NSSI and its associated factors. A cross-cultural study on NSSI and SA among adolescents is also a direction that we will consider in the future.

## Conclusion

Our findings indicate that interventions regarding “limited access to emotion regulation strategies “ theoretically have the potential to alleviate overall network symptoms, enabling adolescents to develop healthier coping mechanisms in their complex environment. Behaviors such as “punching walls, desks, and other hard objects” and “hitting oneself with fists, slaps, or harder objects” frequently co-occur among adolescents who engage in NSSI; as this may indicate an increased risk of future SAs, this should be closely monitored. Furthermore, bridge nodes, such as “personal distress” and “suicidal ideation”, warrant particular attention in clinical settings. Collectively, this study provides a novel network perspective that elucidates the complex interplay between NSSI and SAs in adolescents, highlighting specific targets for prevention and intervention.

## Ethical Approval

This study was approved by the Ethics Committee of the Beijing Huilongguan Hospital (approval number 2021-24-Ke).

## Data Availability

The data that support the findings of this study are available from the corresponding authors upon reasonable request.
