# Supplementary material for: The Role of Limited Emotion Regulation Strategies on Nonsuicidal Self-injury and Suicide Attempts among Chinese Adolescents: A Network Analysis Based on Jiangxi Province
Source: Health Data Sci. 2026 Feb 3;6:0195. doi: 10.34133/hds.0195 (PMC12864651; doi:10.34133/hds.0195)

# Supplementary Materials

# Table S1 Characteristics of 2,496 participants: data shown as N (%) and mean(±SD)

|  | **No SA** | **SA** |  |  |
| --- | --- | --- | --- | --- |
|  | **N=1,921(76.96%)** | **N=575(23.03%)** |  |  |
|  | **N(%) / mean(±SD)** | **N(%) / mean(±SD)** | **t/χ²** | ***p* value** |
| Gender(female) ^a^ | 975(50.80%) | 400(69.60%) | 63.29 | <0.001 |
| Age ^b^ | 15.98(±2.52) | 15.83(±1.34) | 1.37 | 0.17 |
| Height ^b^ | 164.63(±13.24) | 161.85(±15.46) | 4.24 | <0.001 |
| Weight ^b^ | 63.70(±24.74) | 63.54 (±25.69) | 0.13 | 0.90 |
| One-child family ^a^ | 84(4.37%) | 29(5.04%) | 0.46 | 0.50 |
| Order of birth ^a^ |  |  | 6.995 | 0.14 |
| First | 718(37.38%) | 225(39.13%) |  |  |
| Second | 835(43.47%) | 266(46.26%) |  |  |
| Third | 229(11.92%) | 55(9.57%) |  |  |
| Fourth | 91(5.05%) | 18(3.13%) |  |  |
| Fifth | 42(2.19%) | 11(1.91%) |  |  |
| Type of residence ^a^ |  |  | 4.50 | 0.11 |
| Downtown | 203(10.57%) | 66(11.48%) |  |  |
| Suburb | 506(26.34%) | 174(30.26%) |  |  |
| Rural area | 1,212(63.09%) | 335(58.26%) |  |  |
| Way of parenting ^a^ |  |  | 2.03 | 0.73 |
| Ⅰ | 899(46.80%) | 254(44.17%) |  |  |
| Ⅱ | 380(19.78%) | 117(20.35%) |  |  |
| Ⅲ | 413(21.50%) | 135(23.48%) |  |  |
| Ⅳ | 105(5.47%) | 35(6.09%) |  |  |
| Ⅴ | 124(6.46%) | 34(5.91%) |  |  |
| PHQ-9 ^b^ | 8.66(±5.10) | 12.54(±5.97) | -14.12 | <0.001 |
| PHQ-9 1 | 1.14(±0.78) | 1.49(±0.85) | -8.69 | <0.001 |
| PHQ-9 2 | 1.04(±0.77) | 1.49(±0.86) | -11.24 | <0.001 |
| PHQ-9 3 | 1.04(±0.94) | 1.52(±1.01) | -10.05 | <0.001 |
| PHQ-9 4 | 1.17(±0.83) | 1.62(±0.90) | -10.83 | <0.001 |
| PHQ-9 5 | 0.86(±0.84) | 1.28(±0.96) | -9.42 | <0.001 |
| PHQ-9 6 | 1.26(±0.90) | 1.70(±0.95) | -9.86 | <0.001 |
| PHQ-9 7 | 0.79(±0.88) | 1.13(±1.02) | -7.19 | <0.001 |
| PHQ-9 8 | 1.18(±0.94) | 1.18(±0.94) | -10.97 | <0.001 |
| PHQ-9 9 | 0.65(±0.75) | 1.15(±0.94) | -11.51 | <0.001 |
| IRI ^b^ | 89.41(±11.62) | 93.84(±11.53) | -15.80 | <0.001 |
| PT | 22.02(±4.39) | 23.05(±4.38) | -4.94 | <0.001 |
| FS | 22.22(±4.47) | 23.51(±4.29) | -6.15 | <0.001 |
| EC | 21.61(±3.20) | 22.21(±3.27) | -3.89 | <0.001 |
| PD | 22.96(±4.31) | 25.04(±4.11) | -10.25 | <0.001 |
| MPATS ^b^ | 43.83(±12.86) | 50.09(±13.28) | -17.71 | <0.001 |
| withdraw | 17.42(±5.49) | 19.87(±5.71) | -9.33 | <0.001 |
| highlight | 9.05(±3.35) | 10.48(±3.81) | -8.08 | <0.001 |
| social | 8.88(±3.06) | 9.97(±2.98) | -7.51 | <0.001 |
| mood | 8.48(±2.92) | 9.77(±2.94) | -9.28 | <0.001 |
| DERS ^b^ | 92.46(±16.44) | 103.75(±18.09) | -22.41 | <0.001 |
| aware | 19.98(±4.70) | 18.92(±4.87) | 4.72 | <0.001 |
| clarity | 12.98(±2.83) | 14.19(±3.24) | -8.13 | <0.001 |
| non-acceptance | 13.88(±5.06) | 16.76(±5.65) | -10.97 | <0.001 |
| impulse | 14.06(±4.96) | 17.26(±5.63) | -12.29 | <0.001 |
| goals | 14.78(±4.10) | 16.87(±4.27) | -10.36 | <0.001 |
| strategies | 20.67(±6.51) | 25.00(±7.16) | -12.98 | <0.001 |
| NSSI Behavior ^b^ | 5.20(±6.12) | 11.10(±10.28) | -13.07 | <0.001 |
| NSSI 1 | 0.71(±0.90) | 1.34(±1.18) | -11.90 | <0.001 |
| NSSI 2 | 0.59(±0.87) | 1.29(±1.20) | -13.01 | <0.001 |
| NSSI 3 | 0.43(±0.78) | 0.90(±1.14) | -9.24 | <0.001 |
| NSSI 4 | 1.02(±0.94) | 1.40(±1.18) | -7.00 | <0.001 |
| NSSI 5 | 0.42(±0.76) | 0.97(±1.19) | -10.39 | <0.001 |
| NSSI 6 | 0.41(±0.78) | 0.93(±1.16) | -10.00 | <0.001 |
| NSSI 7 | 0.34(±0.74) | 0.65(±1.06) | -6.46 | <0.001 |
| NSSI 8 | 0.26(±0.65) | 0.78(±1.15) | -10.41 | <0.001 |
| NSSI 9 | 0.33(±0.69) | 1.11(±1.23) | -14.57 | <0.001 |
| NSSI 10 | 0.13(±0.48) | 0.35(±0.89) | -5.79 | <0.001 |
| NSSI 11 | 0.25(±0.65) | 0.67(±1.12) | -8.70 | <0.001 |
| NSSI 12 | 0.31(±0.68) | 0.70(±1.11) | -8.09 | <0.001 |

^a^ Chi-squared test. ^b^ t-test. Abbreviations: NSSI, non-suicidal self-injury; SA, suicide attempt; No SA, no suicide attempt; I, living with parents; II, one of the parents is absent periodically; III, living with grandparents without parents; IV, single-parent family; V, other.

# Table S2 Characteristics of 6,959 participants: data shown as N (%) and median (Q1, Q3)

|  | **No SA**  **N=6,209(89.2%)** | **SA**  **N=750(10.8%)** |  |  |
| --- | --- | --- | --- | --- |
|  | **N(%) / median(Q1 ,Q3)** | **N(%) / median(Q1 ,Q3)** | ***χ2/*Z** | ***p*** |
| Gender(female) ^a^ | 3,224(51.92%) | 526(70.13%) | 89.29 | <0.001 |
| Age ^b^ | 16(15, 16) | 16(15, 16) | -2.45 | 0.01 |
| Height ^b^ | 165(160, 170) | 162(158, 168) | -7.37 | <0.001 |
| Weight ^b^ | 54(48, 69) | 53(47, 75) | -1.01 | 0.31 |
| One-child family ^a^ | 301(4.85%) | 34(4.53%) | 0.14 | 0.70 |
| Order of birth ^a^ |  |  | 9.87 | 0.04 |
| First | 2,179(35.09%) | 283(37.73%) |  |  |
| Second | 2,806(45.19%) | 353(47.07%) |  |  |
| Third | 838(13.50%) | 77(10.27%) |  |  |
| Fourth | 272(4.38%) | 23(3.07%) |  |  |
| Fifth | 114(1.84%) | 14(1.87%) |  |  |
| Type of residence ^a^ |  |  | 14.03 | <0.001 |
| Downtown | 600(9.66%) | 89(11.87%) |  |  |
| Suburb | 1,546(24.90%) | 222(29.60%) |  |  |
| Rural area | 4,063(65.44%) | 439(58.53%) |  |  |
| Way of parenting ^a^ |  |  | 10.93 | 0.03 |
| Ⅰ | 3,008(48.45%) | 324(43.20%) |  |  |
| Ⅱ | 1,162(18.71%) | 146(19.47%) |  |  |
| Ⅲ | 1,384(22.29%) | 184(24.53%) |  |  |
| Ⅳ | 305(4.91%) | 52(6.93%) |  |  |
| Ⅴ | 350(5.64%) | 44(5.87%) |  |  |
| NSSI Behavior(binary) ^a^ | 1,921(30.94%) | 575(76.67%) | 608.30 | <0.001 |
| PHQ-9 total ^b^ | 5(2, 9) | 11(7, 16) | -23.69 | <0.001 |
| IRI total ^b^ | 86(78, 94) | 94(87, 101.25) | -15.80 | <0.001 |
| MPATS total ^b^ | 38(29, 47) | 48(39, 57) | -17.71 | <0.001 |
| DERS total ^b^ | 83(75, 92) | 100(88, 114) | -22.41 | <0.001 |
| NSSI Behavior total ^b^ | 0(0, 1) | 5(1, 13) | -29.11 | <0.001 |

^a^ Chi-squared test. ^b^ Mann–Whitney U test. Abbreviations: NSSI, non-suicidal self-injury; SA, suicide attempt; No SA, no suicide attempt; Ⅰ, living with parents; Ⅱ, one of the parents is absent periodically; Ⅲ, living with grandparents without parents; Ⅳ, single parent family; Ⅴ, other.

# Fig. S1 Centrality stability for NSSI-NoSA


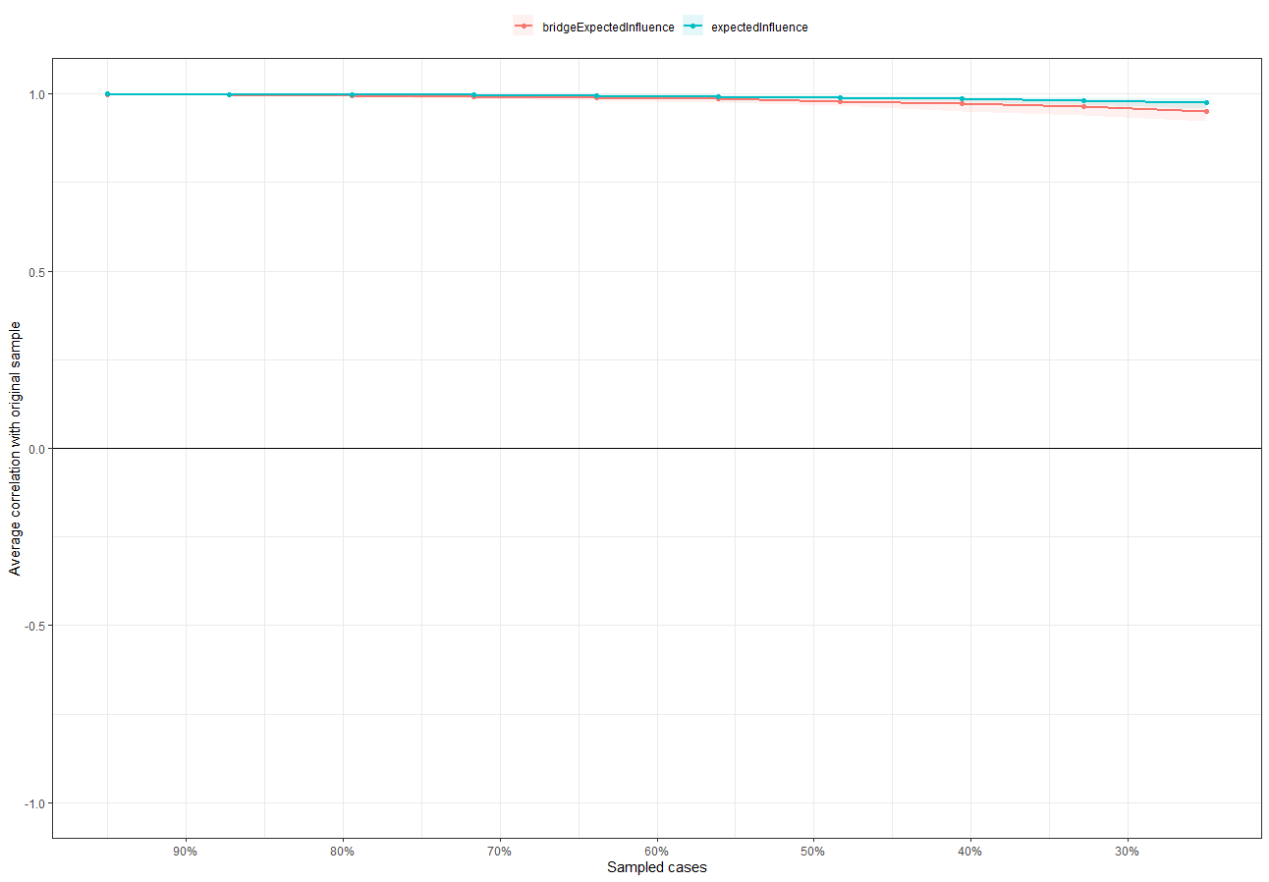


# Fig. S2 Centrality stability for NSSI-SA


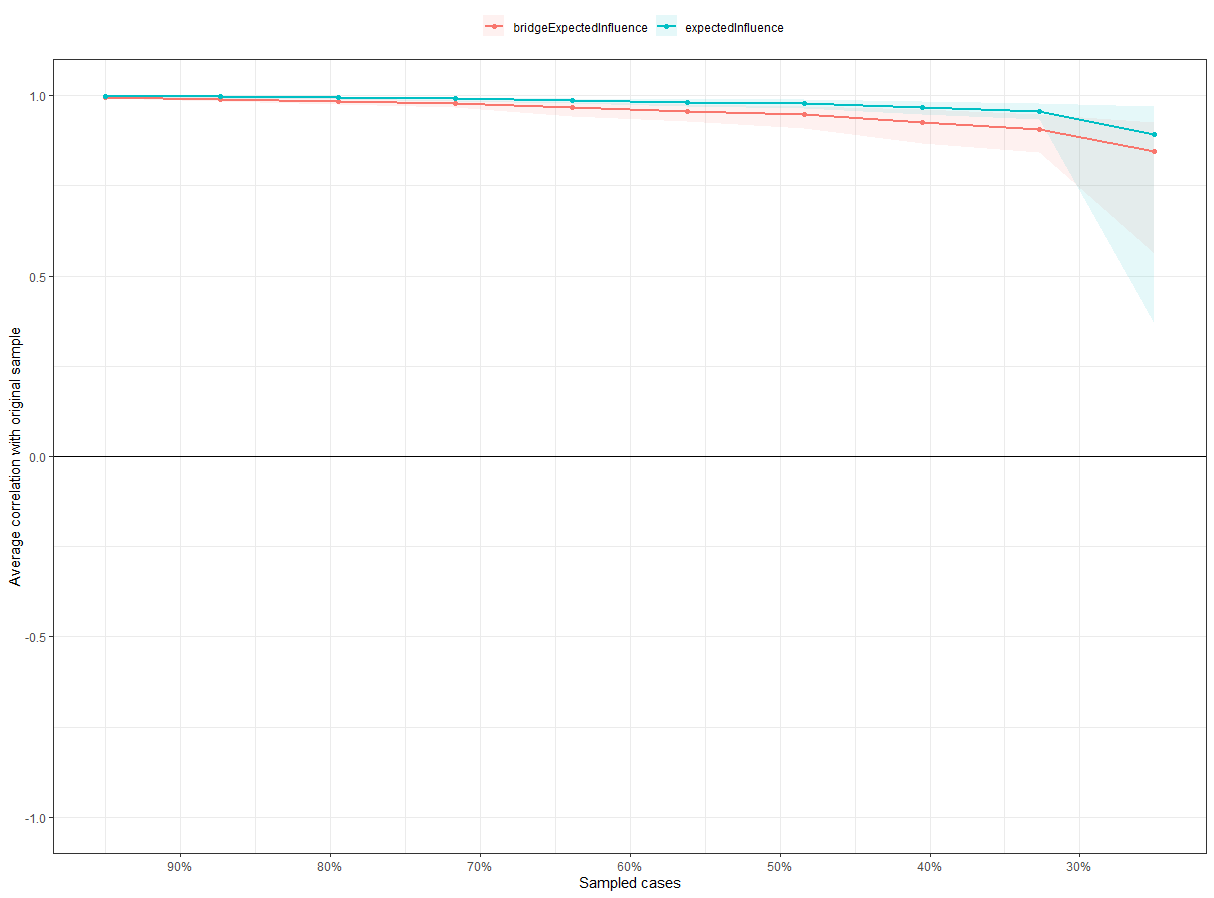


# Fig. S3 Bootstrapped 95% confidence intervals of the edge weights for NSSI-NoSA


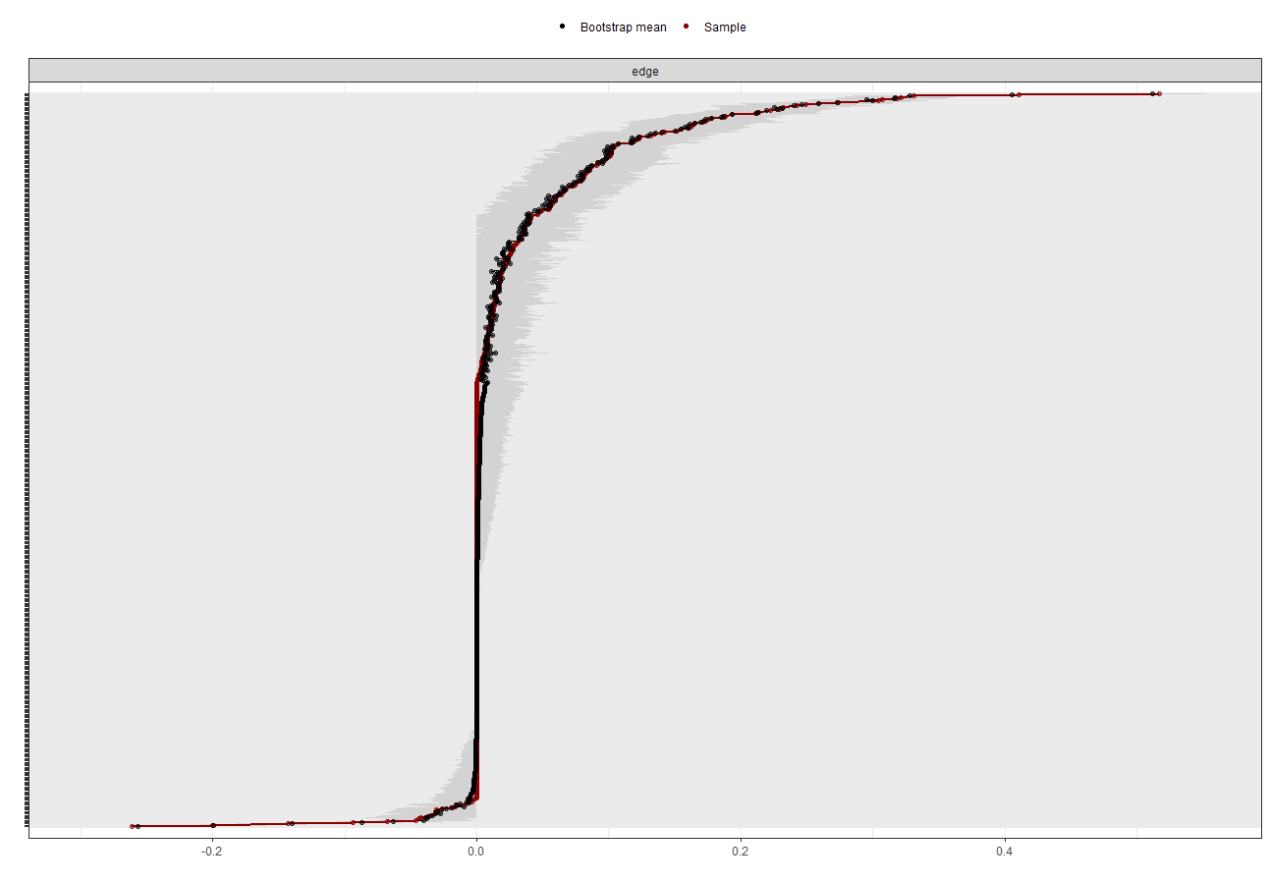


# Fig. S4 Bootstrapped 95% confidence intervals of the edge weights for NSSI-NoSA


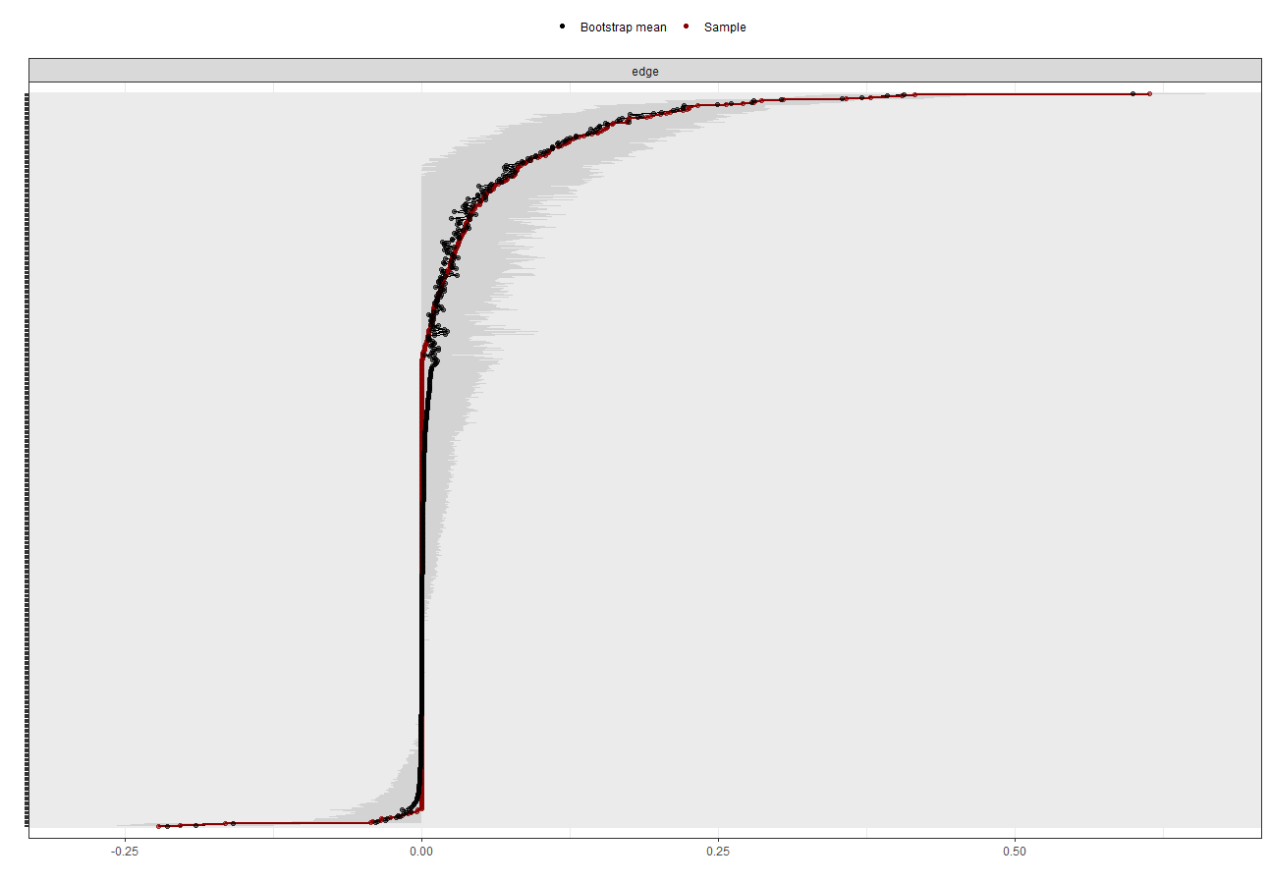


# Fig. S5 Expected influence for NSSI-NoSA


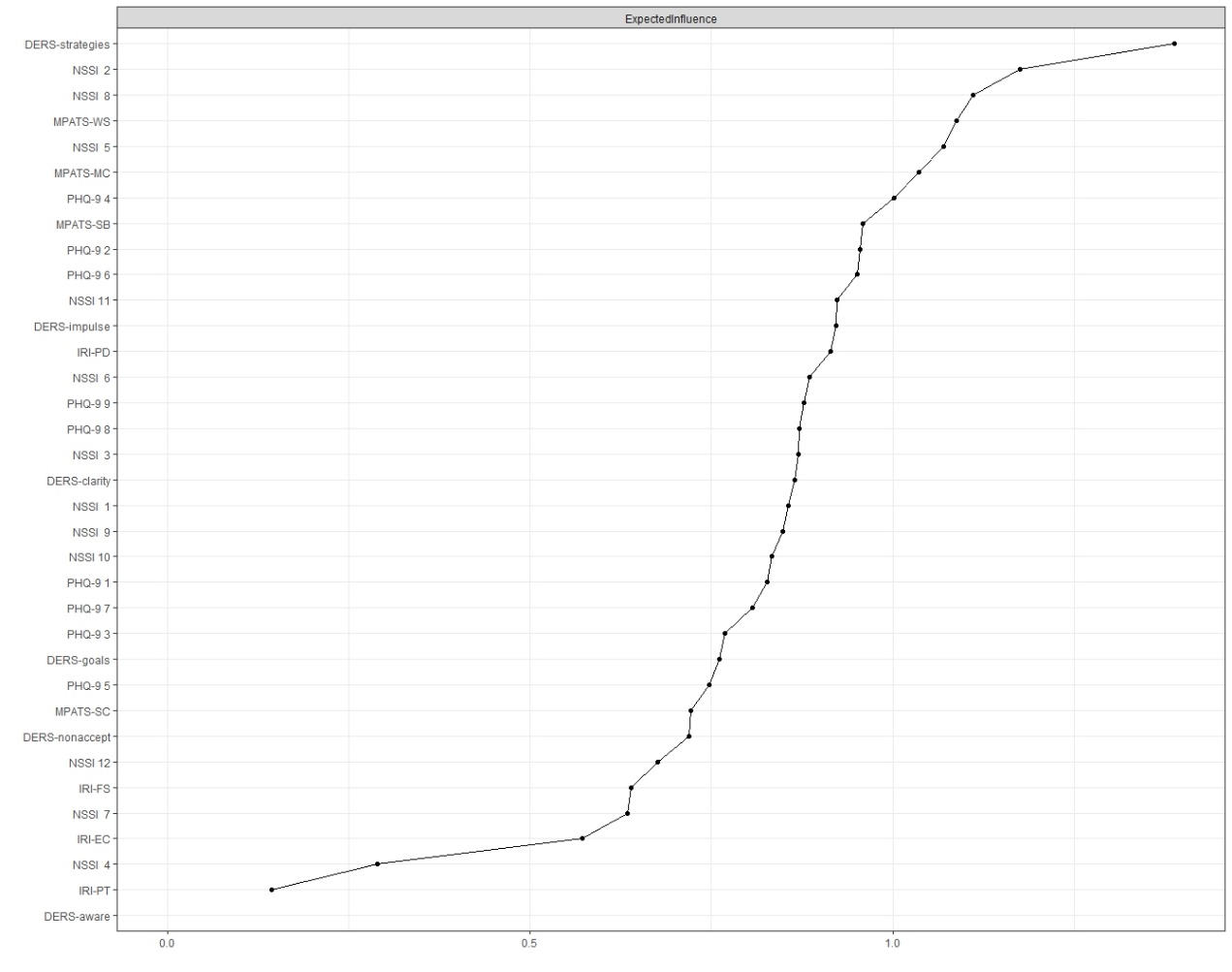


# Fig. S6 Expected influence for NSSI-SA


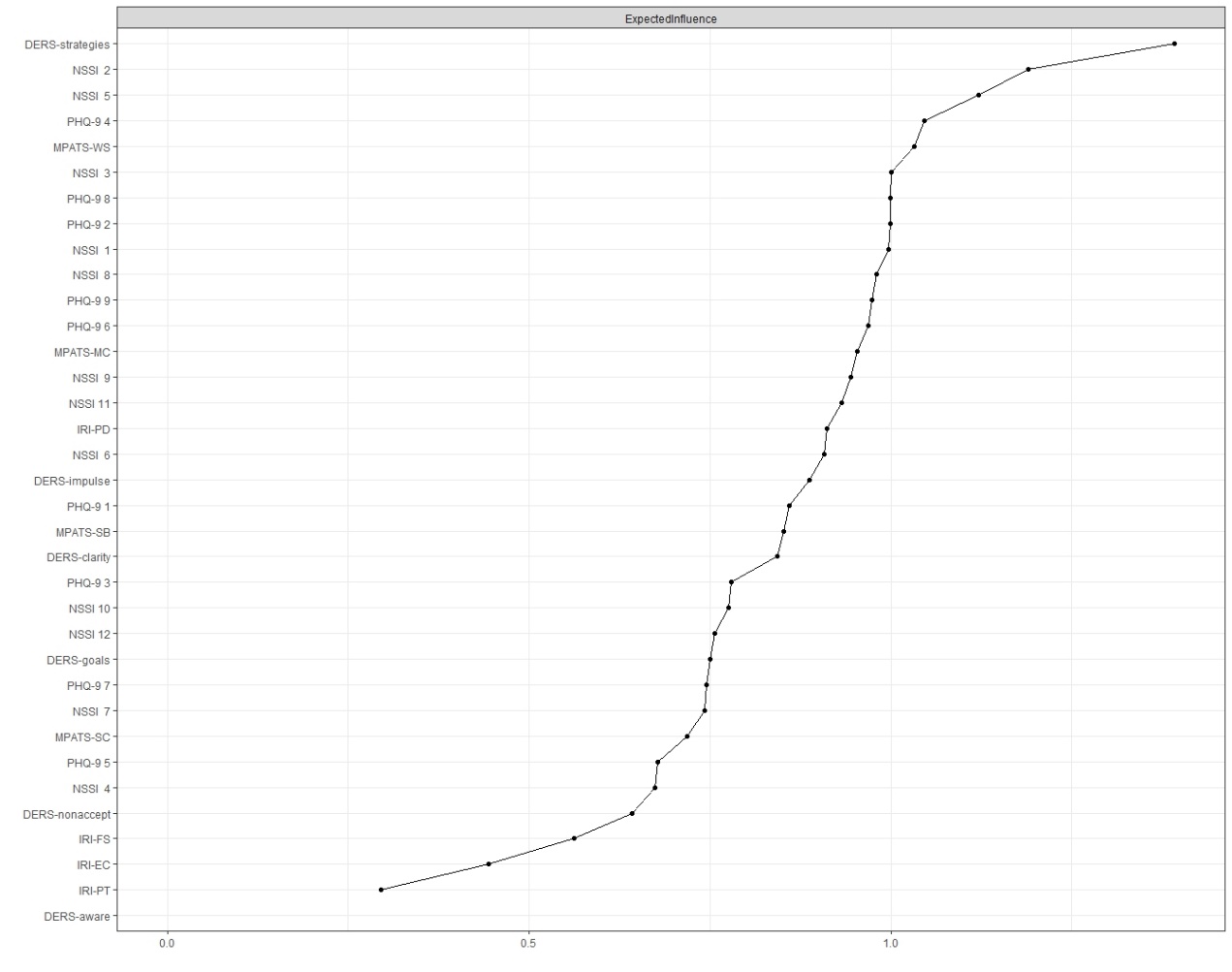

Supplement: Supplementary 1 — Figs. S1 to S6 Tables S1 and S2 [file hds.0195.f1.doc]
